# Opioid Use Is Not Associated with Incomplete Wireless Capsule Endoscopy for Inpatient or Outpatient Procedures

**DOI:** 10.1155/2014/651259

**Published:** 2014-08-19

**Authors:** Bryan Kleinman, Peter P. Stanich, Kavita Betkerur, Kyle Porter, Marty M. Meyer

**Affiliations:** ^1^Department of Internal Medicine, The Ohio State University Wexner Medical Center, Columbus, 43210 OH, USA; ^2^Division of Gastroenterology, Einstein Medical Center, Philadelphia, 19141 PA, USA; ^3^Section of Capsule Endoscopy, Division of Gastroenterology, Hepatology & Nutrition, The Ohio State University Wexner Medical Center, Columbus, 43210 OH, USA; ^4^College of Medicine, The Ohio State University Wexner Medical Center, Columbus, 43210 OH, USA; ^5^Center for Biostatistics, The Ohio State University Wexner Medical Center, Columbus, 43210 OH, USA

## Abstract

*Objective*. Wireless capsule endoscopy (WCE) is commonly used to directly visualize the small bowel. Opioids have variably been linked with incomplete studies and prolonged transit times in heterogeneous cohorts. We aimed to investigate the effect of opioid use on WCE for inpatient and outpatient cohorts. *Methods*. We performed a retrospective review of patients receiving WCE at our institution from April 2010 to March 2013. Demographic data, medical history, and WCE details were collected. Transit times were compared by log-rank analysis. Multivariable logistic regression and Cox proportional hazard models were utilized. *Results*. We performed 314 outpatient and 280 inpatient WCE that met study criteria. In the outpatient cohort, gastric transit time (GTT) was not significantly different between opioid and nonopioid users. Completion rates were similar as well (88% and 87%, *P* = 0.91). In the inpatient cohort, GTT was significantly longer in patients receiving opioids than in patients not receiving opioids (44 versus 23 min, *P* = 0.04), but completion rates were similar (71% versus 75%, *P* = 0.31). *Conclusion*. Opioid use within 24 hours of WCE did not significantly affect completion rates for inpatients or outpatients. GTT was prolonged in inpatients receiving opioids but not in outpatients.

## 1. Introduction

Wireless capsule endoscopy has become an increasingly popular method to image the small bowel since its introduction in 2000 [1]. WCE is currently used for many indications and can be performed in both the inpatient and outpatient settings. Despite the increased use of WCE, there are still a significant number of incomplete studies where the WCE does not reach the cecum during the battery lifespan. The current literature suggests that WCE completion is only accomplished in 83.5% of studies [2].

Multiple previous studies have identified inpatient capsule endoscopy as having a higher rate of incomplete studies as compared to outpatient procedures [3–6]. Other reported risk factors for incomplete studies include medications, systemic medical conditions, immobility, and previous small bowel surgery [3–5, 7]. To our knowledge, there has not been a large scale study to date looking at the effect of opioids on WCE transit times and completion rates. With the increasing rates of opioid use, this information would be of benefit to centers that perform WCE.

The aim of the current study was to investigate the effects of medications, specifically opioids, and other systemic medical conditions on WCE transit times and completion rates in both inpatients and outpatients at a tertiary care academic medical center.

## 2. Materials and Methods

### 2.1. Patients

The appropriate institutional review board approved this study prior to initiation. A database of all patients undergoing WCE at our institution from April 2010 to March 2013 was utilized to identify potential study subjects. The database was compiled retrospectively and included demographic data and pertinent clinical history. Details regarding the WCE including transit times, findings, and completion status were obtained from the report created at the time of interpretation. We recorded pertinent medication use based on a preset list of medications with known effects on gastrointestinal motility. We also recorded body mass index, medical conditions with effects on gastrointestinal motility, and the mobility of the patient. Gastric transit time (GTT) was calculated by the time that separated the first image of stomach from the first image of duodenum. Small bowel transit time (SBTT) was calculated as the time that separated the first image of duodenum from the first image of cecum. Total transit time (TTT) was considered as the combination of these measures. An examination was considered to be complete when the cecum was reached during the battery lifespan. Capsule retention was defined as the device remaining in the bowel at least 14 days after ingestion. A WCE was considered to be diagnostic when a finding provided an explanation for the stated indication of the procedure.

Inclusion criteria for the current study were patients 18 years of age or greater undergoing inpatient or outpatient WCE at our institution. We excluded patients who had the WCE placed endoscopically or if their medications were not included in the medical record.

### 2.2. Procedure

Our standard recommendations for patients scheduled to receive a WCE study were to remain NPO for at least 8 hours prior to the study. A 2 L polyethylene glycol bowel preparation was ordered for the evening before the procedure. Clear liquid intake and then light food intake were allowed at 2 and 4 hours postingestion, respectively. No prokinetic medications were utilized. All WCE during the study period were PillCam Small-Bowel 2 Video Capsules (Given Imaging, Yoqneam, Israel) and were interpreted with the associated software by either of 2 experienced physicians (including author MMM).

### 2.3. Statistical Analysis

Patient characteristics and clinical data were presented as means with standard deviations for continuous variables and as counts and percentages for categorical variables. Median and interquartile range (IQR) were used to summarize gastric, small bowel, and total transit times. These transit times were compared using multivariable Cox proportional hazards models, with the end of battery life used to censor incomplete procedures. Capsule completion was analyzed by multivariable logistic regression. In all multivariable models, covariates included opioid use and any patient characteristic with a *P* value of 0.1 or less in group comparisons.

## 3. Results

594 patients met study criteria during the specified time period (314 outpatients and 280 inpatients). Demographic data is included in Table 1. Of the 314 outpatient studies, 89 (28%) patients had opioid use within 24 hours of the procedure. There was an increased incidence of female patients (*P* = 0.02), prior bariatric surgery (*P* = 0.03), and Crohn's disease (*P* = 0.002) in the group receiving opioids. They also had a higher proportion of patency capsule prior to the WCE (*P* = 0.002). There were a significantly higher number of patients in the opioid group that had received anticholinergic medications within 24 hours of the WCE (43% versus 21%, *P* < 0.001). Demographic data and medical history were otherwise similar.

**Figure 1:**
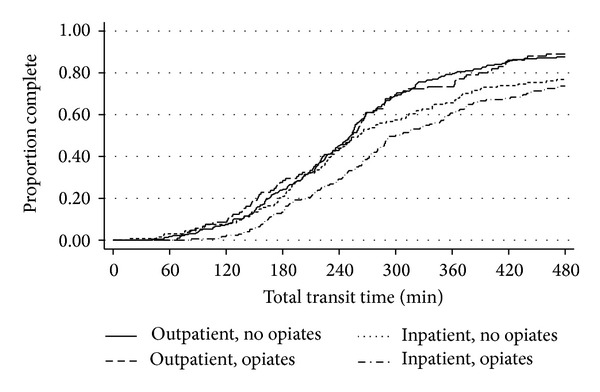
Kaplan-Meier analysis of total transit time and video capsule completionin inpatients and outpatients with and without opioids within 24 hours of the study. Statistical analysis showed no significant difference in completion rates in inpatients with/without opioid use (*P* = 0.31) and outpatients with/without opioid use (*P* = 0.91).

After multivariable analysis adjusting for the differences in the cohorts, outpatients without opioids had a median TTT of 252 minutes (IQR 185–323) as compared to 261 minutes (IQR 183–363) in patients that received opioids (*P* = 0.95) (Table 2). GTT was also similar between the two groups with a median of 25 minutes (IQR 14–54) in the group not receiving opioids within 24 hours as compared to 24 minutes (IQR 10–69) in the group receiving opioids (*P* = 0.53). Completion rates were similar between the groups (88% and 87%, resp., *P* = 0.91, Figure 1). There were similar rates of gastric capsule retention. There remained no significant differences between opioid and nonopioid users in these measures when opioid use at the 48-hour and 7-day time points was considered.

Of the 280 inpatient studies, 147 patients (52.5%) had used opioids within 24 hours of the WCE. Patients in the nonopioid cohort were older (65.3 versus 59.3 years, *P* < 0.001) and more likely to have diabetes (*P* = 0.02). The opioid users received the WCE on an earlier hospital day than the nonopioid group (5.1 days and 6.3 days, resp., *P* = 0.02) and there was a trend toward shorter hospitalization in the opioid group (10.0 days and 11.8 days, resp., *P* = 0.08). A significantly higher proportion of the nonopioid users had received beta-blockers within 24 hours of the WCE (65% versus 46%, *P* = 0.001). Demographic data and medical history were otherwise similar in the cohorts.

After multivariable analysis adjusting for the differences in the cohorts, GTT was significantly longer in patients using opioids (44 versus 23 min, *P* = 0.04, Table 2). There was a trend towards the number of GTT >45 minutes in the group receiving opioids (48% versus 35%, *P* = 0.09). TTT was neither significantly increased in patients receiving opioids (300 versus 260 min, *P* = 0.11) nor was there a significant difference in completed studies between the two groups (71% versus 75%, *P* = 0.31). Gastric retention rates were similar in the cohorts. There remained no significant differences when opioid use at 48 hours and 7 days was considered.

## 4. Discussion

WCE has become an increasingly popular method to evaluate the small bowel since its introduction to the market. With the increased use, incomplete studies are a common occurrence. In this study, we aimed to identify if opioid use was a specific risk factor for incomplete studies when inpatient and outpatient procedures were considered separately given the previously identified difference in performance characteristics in these groups [3–6].

Inpatient WCE studies in our cohort had a higher rate of incompletion as compared to outpatients, as well as when compared to the completion rate reported in the literature [2]. In our inpatient cohort, we found gastric transit time was significantly longer in patients receiving opioids within 24 hours as compared to patients not receiving opioids. Despite this prolonged gastric transit time, there was no significant difference between total transit times and completion rates between the two groups. Although prolonged gastric transit time has been found to be associated with incomplete WCE, a GTT of greater than 45 minutes has been utilized for this demarcation [5]. One reason for our findings may have been that both groups remained below this marker. Diabetes mellitus has previously been found to be a risk factor for prolonged GTT and incomplete studies [7]. Despite the fact that there were more diabetics in the inpatient nonopioid cohort, this was corrected for within the multivariable analyses and thus should not affect the findings. Based on the results in the inpatient cohort, we do not find compelling evidence to withhold opioids in patients that are hospitalized and receiving WCE.

Furthermore, we also did not find any significant differences in the outpatient setting when we compared patients with and without opioid use within 24 hours of the study. The completion rates in both outpatient groups were higher than the inpatient groups and also were in line with current reported completion rates. As a result of these similarities, we do not feel it is necessary to make any changes to a stable outpatient medication regimen.

The primary limitations of our study are the cohort size and retrospective nature. It should be noted, however, that our cohorts are of a relatively large sample size when compared to previously published literature. A prospective study should be considered in the future as it may be helpful to look closely at opioid dosing and the effects on transit times and completion rates. Specifically, comparison of the effects of high versus low daily opioid intake and acute and chronic use would be of interest. Our retrospective design limited our ability to discern this.

In summary, we found that inpatients receiving opioids within 24 hours of WCE had prolonged gastric transit times but no significant differences with total transit times and completion rates in comparison to inpatients not receiving opioids. In outpatients, we found no significant differences in transit times or completion rates in patients using opioids as compared to patients not using opioids within 24 hours.

## Figures and Tables

**Table 1:** Demographics, clinical characteristics, and medication usage of patients receiving WCE grouped by opioid use and location of procedure.

	Inpatients			Outpatients		
	No opioids 24 hours	Opioids 24 hours	*P* value∗	No opioids 24 hours	Opioids 24 hours	*P* value∗
Patients	*n* = 133	*n* = 147		*n* = 225	*n* = 89	
Sex (female), *n* (%)	78 (59%)	90 (61%)	0.61	133 (59%)	64 (73%)	0.02
Age, mean (SD)	65.3 (13.3)	59.3 (15.1)	<0.001	52.6 (17.7)	50.0 (13.9)	0.23
Body mass index, mean (SD)	30.7 (8.1)	29.2 (7.9)	0.23	30.6 (28.6)	29.5 (9.1)	0.52
Bowel preparation, *n* (%)	133 (100%)	145 (99%)	0.5	220 (99%)	88 (99%)	0.5
Poor preparation, *n* (%)	11 (8%)	13 (9%)	0.86	16 (7%)	9 (10%)	0.39
Patency capsule performed, *n* (%)	27 (20%)	17 (12%)	0.045	35 (16%)	28 (31%)	0.002
Total hospital days, *n* (SD)	11.8 (8.7)	10.0 (7.5)	0.08			
WCE hospital day, *n* (SD)	6.3 (4.5)	5.1 (4.0)	0.02			
Clinical characteristics:						
Crohn's disease, *n* (%)	4 (3%)	11 (7%)	0.1	14 (6%)	16 (18%)	0.002
Diabetes mellitus, *n* (%)	68 (51%)	54 (37%)	0.02	46 (21%)	18 (20%)	0.92
Hypothyroidism, *n* (%)	29 (22%)	22 (15%)	0.14	33 (15%)	15 (17%)	0.68
Small bowel surgery, *n* (%)	17 (13%)	26 (18%)	0.26	12 (5%)	10 (11%)	0.07
Bariatric surgery, *n* (%)	5 (4%)	6 (4%)	0.89	4 (2%)	6 (7%)	0.03
History of bowel obstruction, *n* (%)	3 (2%)	8 (5%)	0.17	6 (3%)	5 (6%)	0.2
Medications:						
Prokinetics, *n* (%)	6 (5%)	10 (7%)	0.41	2 (1%)	3 (3%)	0.11
Beta-blockers, *n* (%)	87 (65%)	67 (46%)	0.001	63 (28%)	34 (38%)	0.08
Calcium channel blockers, *n* (%)	29 (22%)	23 (16%)	0.19	20 (9%)	11 (12%)	0.36
Anticholinergics, *n* (%)	13 (10%)	26 (18%)	0.06	46 (21%)	38 (43%)	<0.001
Iron supplementation, *n* (%)	37 (28%)	35 (24%)	0.44	76 (34%)	27 (30%)	0.56

∗Chi-square or Fisher's exact tests for categorical variables and *t*-tests or Mann-Whitney *U* test for continuous variables.

**Table 2:** WCE transit times and completion rates grouped by opioid use and location of procedure.

	Inpatients			Outpatients		
	No opioids 24 hours	Opioids 24 hours	*P* value^1^	No opioids 24 hours	Opioids 24 hours	*P* value^2^
GTT, min., median (IQR)	23 (8, 64)	44 (14, 87)	0.04	25 (14, 54)	24 (10, 69)	0.53
SBTT, min., median (IQR)	211 (159, 351)	240 (171, 349)	0.62	213 (142, 279)	206 (125, 305)	0.87
TTT, min., median (IQR)	260 (189, 460)	300 (223, >480)	0.11	252 (185, 323)	261 (183, 363)	0.95
Completed exam, *n* (%)	100 (75%)	104 (71%)	0.31	197 (88%)	77 (87%)	0.91
Gastric capsule retention, *n* (%)	5 (4%)	9 (6%)	0.24	9 (4%)	3 (3%)	0.93

^1^Multivariable Cox proportion hazards model (transit times) or multivariable logistic regression model (completion, retention) adjusting for age, history of diabetes, history of Crohn's, and hospital length of stay.

^2^Multivariable Cox proportion hazards model (transit times) or multivariable logistic regression model (completion, retention) adjusting for sex, history of Crohn's, and prior small bowel surgery.

GTT, gastric transit time; IQR, interquartile range; min., minutes; SBTT, small bowel transit time; TTT, total transit time.

## Abbreviations

CI: Confidence interval
GTT: Gastric transit time
IQR: Interquartile range
OR: Odds ratio
SBTT: Small bowel transit time
TTT: Total transit time
WCE: Wireless capsule endoscopy.
